# The “Mevalonate hypothesis”: a cholesterol-independent alternative for the etiology of atherosclerosis

**DOI:** 10.1186/1476-511X-11-149

**Published:** 2012-11-05

**Authors:** Hiskias G Keizer

**Affiliations:** 1 Stepan Specialty Products B.V., Museumlaan 16, 1541 LP, Koog aan de Zaan, The Netherlands

## Abstract

The “cholesterol hypothesis” is the leading theory to explain the cause of atherosclerosis. The “cholesterol hypothesis” assumes that plasma (LDL) cholesterol is an important causal factor for atherosclerosis.

However, data of at least seven placebo controlled randomized prospective trials with various cholesterol lowering drugs show that plasma cholesterol lowering does not necessarily lead to protection against cardiovascular disease. Therefore an alternative hypothesis for the etiology of cardiovascular disease is formulated. This alternative hypothesis, the “mevalonate hypothesis”, assumes that after stimulation of the mevalonate pathway in endothelial cells by inflammatory factors, these cells start producing cholesterol and free radicals. In this hypothesis, only the latter play a role in the etiology of atherosclerosis by contributing to the formation of oxidized cholesterol which is a widely accepted causal factor for atherosclerosis.

Regardless of how the mevalonate pathway is activated (by withdrawal of statin drugs, by inflammatory factors or indirectly by reduced intracellular cholesterol levels) in all these cases free radical production is observed as well as cardiovascular disease. Since in the “mevalonate hypothesis” cholesterol is produced at the same time as the free radicals causing atherosclerosis, this hypothesis provides an explanation for the correlation which exists between cardiovascular disease and plasma cholesterol levels. From an evolutionary perspective, concomitant cholesterol production and free radical production in response to inflammatory factors makes sense if one realizes that both activities potentially protect cells and organisms from infection by gram-negative bacteria.

In conclusion, data have been collected which suggest that activation of the mevalonate pathway in endothelial cells is likely to be a causal factor for atherosclerosis. This “mevalonate hypothesis” provides a better explanation for results obtained from recent clinical studies with cholesterol lowering drugs than the “cholesterol hypothesis”. Furthermore, this hypothesis explains how cholesterol can be correlated with cardiovascular disease without being a causal factor for it. Finally it provides a logical explanation for the etiology of this disease.

## Introduction

In a series of review articles [1-5], Daniel Steinberg explains how the world got convinced of the “cholesterol hypothesis”. This hypothesis states that plasma cholesterol is a causal factor for atherosclerosis. Authorities, including the European Food and Safety Authority (EFSA), appear to accept the “cholesterol hypothesis” as they approved health claims for certain ingredients in the area of cardiovascular health based on the sole fact that those ingredients lower plasma cholesterol values. Also, well known scientists in the field of nutrition reason that functional foods which increase plasma cholesterol levels can be dangerous for the cardiovascular system [6].

However, there have been contradictory observations in the recent years regarding the role of plasma cholesterol in cardiovascular disease, and this was the reason to further look into the actual evidence that plasma cholesterol would be the major causal factor for cardiovascular disease. In order to do this, evidence concerning the cholesterol dependent mechanisms for atherosclerosis were closely evaluated as well as pharmacological evidence obtained from trials on the efficacy of cholesterol lowering drugs. Also, epidemiological evidence for the relationship between plasma cholesterol and cardiovascular disease was taken into account. Ultimately, an alternative hypothesis was presented which explains why cholesterol is related to cardiovascular disease without being the cause of it.

### The “cholesterol hypothesis”

To be able to understand the “cholesterol hypothesis” it is useful to go back to the evidence behind this hypothesis. Already in 1901, Windaus discovered that aortas of patients with atherosclerosis contain more cholesterol than aortas of healthy people [7]. Not long thereafter in 1913, Anitschkow showed that feeding cholesterol to rabbits increased their plasma cholesterol and caused atherosclerosis [8]. In the years thereafter, he established that the process of atherosclerosis starts with the formation of fatty streaks. Fatty streaks consist of white blood cells which have infiltrated the arterial wall. Most lipids in the cells of the fatty streaks are contained in foam cells. Later work of Anitschkow showed that these fatty streaks further develop into advanced lesions containing connective tissue. These lesions show high similarity to early atherosclerotic lesions in humans. In 1939, Muller discovered that familial cases of hypercholesterolemia (FH) exist and that these people suffer much more from cardiovascular disease than people without increased plasma cholesterol levels [9], suggesting that plasma cholesterol could be a causal factor in atherosclerosis.

In 1950, Gofman showed that most of the cholesterol in FH patients resided in LDL and IDL fractions. He also showed that there was a correlation between the levels of these lipoproteins in the blood and cardiovascular disease [10].This was the starting point of numerous successful studies done later to confirm the positive correlation between LDL cholesterol and cardiovascular disease.

Not much later in 1951, Barr et al discovered that another cholesterol containing lipoprotein, HDL, was negatively related to cardiovascular disease [11]. This is relevant in view of later discoveries which clarified that cellular uptake of cholesterol in tissues is mediated by receptors which recognize LDL-cholesterol [12], whereas reverse transport of cholesterol to the liver is mediated by HDL cholesterol [13]. Since in patients with cardiovascular disease, plasma LDL-cholesterol levels were increased and plasma HDL-cholesterol levels were decreased, there appeared to be an increased transport of cholesterol into the cardiovascular lesions and a reduced transport of cholesterol from the lesions back to the liver in patients with this disease.

In 1961, the first data of the Framingham study were presented. This study showed for the first time a clear relationship between moderately increased levels of plasma cholesterol and the occurrence of cardiovascular disease [14]. In order to prove that plasma cholesterol was causally involved in cardiovascular disease, it was necessary to show that a decrease in plasma cholesterol would result in a decrease in cardiovascular disease. In 1952, Kinsell had shown that saturated fats increase plasma cholesterol and that polyunsaturated fats decrease plasma cholesterol [15]. One of the first long term studies which indicated that such a cholesterol lowering diet would reduce cardiovascular disease was performed by Leren in 1966 [16]. This positive trial added to the hypothesis that plasma cholesterol can cause cardiovascular disease.

The 7 countries study, published in 1980, showed that the lower the mean plasma cholesterol levels of the population, the lower the cardiovascular death incidence in countries [17]. This further added to that view, although the author had only included countries in this evaluation which supported this hypothesis. In 1984, the now famous LRC CPPT trial was published [18]. This trial showed that cholestyramine, a drug which lowered plasma cholesterol by about 10% by binding cholesterol inside the intestinal tract, lowered the relative risk of coronary heart disease by almost 20%. Although the statistical evaluation of this study was subject to discussion, this result supported the hypothesis that plasma cholesterol can cause cardiovascular disease. Ten years later the well-known 4S study was published [19]. In this study simvastatin was used to reduce plasma cholesterol levels. Simvastatin is an inhibitor of HMGCoA reductase, which is the rate limiting enzyme of the mevalonate pathway which is responsible for the formation of cholesterol. In this study plasma cholesterol was decreased by about 25% and reduced the relative risk of death due to coronary heart disease by 42%.

Together these data made Steinberg to conclude that “the Cholesterol controversy” had ended [5] as in his view plasma cholesterol had been proven to play an important causal role in cardiovascular disease. Many pharmaceutical companies, food companies, university scientists and regulatory authorities followed that view. However, while these arguments seem convincing, several issues remain unresolved. These will be discussed below.

### Doubts on the “cholesterol hypothesis”

In familial hypercholesterolemia (FH) patients have high plasma cholesterol levels and suffer frequently from cardiovascular disease. This may suggests that high plasma cholesterol levels are directly involved in formation of cholesterol loaded foam cells. However, cells of FH patients are defective in cholesterol receptors which in controls are involved in cellular uptake of cholesterol, so increased uptake of native cholesterol due to higher cholesterol concentrations plays no role in the formation of foam cells. Alternatively, it is now widely accepted that macrophages, as predecessors of foam cells in fatty streaks, do not take up native cholesterol but mainly oxidatively modified LDL cholesterol (ox-LDL) using scavenger receptors [3]. For FH this suggests that not the uptake of native cholesterol in macrophages is increased but the uptake of oxidatively modified cholesterol. Apparently oxidatively modified cholesterol, and not native cholesterol, plays an important role in the induction of atherosclerosis.

Steinberg agrees that ox-LDL plays an important role in the etiology of atherosclerosis. However, in the “cholesterol-hypothesis” the relationship between increased plasma cholesterol levels and increased levels of ox-LDL is not entirely clear. It has been argued that in the reaction of LDL-cholesterol with oxygen radicals, plasma LDL cholesterol would be the driving force for the production of oxidized cholesterol. However, this is not very likely: In the reaction between LDL cholesterol and oxygen radicals, oxygen radical concentrations are very low due to their high reactivity, whereas LDL-cholesterol levels in plasma are very high. Under those conditions, pseudo first order kinetics occur. This means that the rate of formation of ox-LDL is determined by the rate of formation of oxygen radicals and not by the concentration of LDL cholesterol since this concentration hardly changes during this reaction. Therefore free radicals are more likely to be a causal factor for atherosclerosis than increased plasma levels of native LDL cholesterol.

Data obtained in a meta-analysis comprising almost 300,000 people showed that HDL cholesterol does not protect against cardiovascular disease [20], and also people with a genetically lowered HDL cholesterol level did not suffer from increased incidences of myocardial infarction [21]. These data undermine the arguments for a protective role of HDL cholesterol in atherosclerosis. Of course it is useless to deny the existence of an inverse correlation between the incidence of cardiovascular disease and plasma HDL cholesterol levels. The data presented in references 20 and 21 do not deny this inverse correlation but just indicate that this inverse correlation does not involve any causality in relation to cardiovascular disease.

The arguments above seriously question a causal role of native LDL cholesterol and HDL cholesterol in atherosclerosis. Further evidence that neither LDL nor HDL cholesterol plays an important role in the development of atherosclerosis comes from several large, randomized, placebo controlled trials which show that various drugs which considerably reduce plasma LDL cholesterol and/or increase plasma HDL cholesterol do not protect against atherosclerosis or cardiovascular disease:

In a trial testing the effect of hormone replacement therapy (HRT) on cardiovascular disease, 2763 women with coronary disease were included [22]. Patients were followed for more than 4 years after having started HRT or placebo treatment. HRT treatment resulted in a decrease of LDL cholesterol levels by 11% and an increase of HDL cholesterol levels by 10%. Despite these “favorable” changes in plasma cholesterol, the overall rate of cardiovascular events did not change.

In a trial testing the effect of torcetrapib, a cholesterol ester transfer protein inhibitor, 850 patients with familial hypercholesterolemia were included [23]. Patients were treated with statins or with statins plus torcetrapib for 2 years. Thereafter carotid intima-media thickness for the common carotic artery was measured as a surrogate marker for atherosclerosis. Treatment with torcetrapib decreased LDL cholesterol levels by about 25% and increased HDL cholesterol by about 35% compared to treatment by statins alone. Despite these “favorable” changes in plasma cholesterol, treatment with torcetrapib/statin resulted in an annual increase of the carotid intima-media thickness, whereas for the statin-only group a small decrease was reported. Torcetrapib therefore appeared to worsen atherosclerosis in this study.

Another example concerns a study with ezetimibe. Ezetimibe inhibits intestinal cholesterol uptake. In this study, 720 patients with familial hypercholesterolemia were included [24]. Patients were treated with statins or with statins plus ezetimibe for 2 years. Thereafter carotid intima-media thickness for the common carotic artery was measured as a surrogate marker for atherosclerosis. Treatment with ezetimibe decreased LDL cholesterol levels by 16.5%. HDL cholesterol did not change. Again these changes in plasma cholesterol after treatment with ezetimibe/statin did not result in positive effects on the intima thickness compared to the statin-only group.

In conclusion, it was shown that unbalanced native cholesterol transport to atherosclerotic lesions is unlikely to play a role in atherosclerosis. This view was confirmed by the results of clinical studies on atherosclerosis using various cholesterol modifying drugs [22-24]. Together these data represent serious doubts that plasma cholesterol has a causal role in atherosclerosis.

### The effect of statins on cardiovascular disease

The protective effects of statins against cardiovascular disease is seen by Steinberg as final proof for the “cholesterol hypothesis” [5]. Statins inhibit HMGCoA reductase activity and thereby reduce plasma cholesterol levels. However, not all trials with statins show protection against cardiovascular disease even though in all these trials clear reductions in plasma LDL cholesterol were achieved [25-28]. These data confirm data of studies with other cholesterol lowering drugs [22-24] that LDL cholesterol is unlikely to be an important causal factor for cardiovascular disease.

At least two different reasons can be considered as explanation for the failure of statins in recent trials: 1) Statins may also have pharmacological effects which may reduce its protective effect and 2) Possibly not all subpopulations are responders to statin treatment.

1. *Statins may have pharmacological effects which reduce its protective effect*. Due to HMGCoA reductase inhibition, which is a rate limiting enzyme of the of the mevalonate pathway, statins have many downstream biological effects. One of those is the inhibition of the formation of Coenzyme Q10 [29]. This is highly relevant as coenzyme Q10 is a major anti-oxidant for circulating LDL cholesterol [30], and many cardiac patients are known to have low circulating Coenzyme Q10 levels [31]. A further reduction of Coenzyme Q10 in such patients may have a more negative impact on atherosclerosis than in patients with a proficient antioxidant system. Also, kidney patients and diabetics suffer from oxidative stress. It is therefore quite possible that plasma Coenzyme Q10 lowering due to statin treatment contributed to the failure of several recent statin trials in such patient groups [25-28].

2. *Not all subpopulations of patients are responders to statin treatment*. Statins are probably most efficacious in patients with an overactive HMGCoA reductase system. An elegant study of Miettienen et al [32,33] actually confirmed that the efficacy of statin treatment is indeed linked to reduction of an overactive HMGCoA reductase system. He divided a group of sufferers from cardiovascular disease who had been treated with statins in four quartiles with increasing cholesterol production. After 5 years of treatment, the effect of statins on protection against cardiovascular disease was compared in the two most extreme different quartiles: Only the group with increased cholesterol synthesis showed reduced frequency of cardiovascular disease on statins.

Together these data would suggest that not plasma cholesterol, but an increased activity of the mevalonate pathway, is an important causal factor for atherosclerosis.

Steinberg concluded that the clinical data obtained with statins had ended “the cholesterol controversy” and that these data prove that plasma cholesterol is a causal factor for atherosclerosis [5]. However, the recent failed studies with statins [25-28] appear to prove the opposite: Plasma cholesterol is unlikely to play a causal role in atherosclerosis. The increased activity of HMGCoa reductase enzyme activity appears to be a more likely causal factor for atherosclerosis.

### The “mevalonate hypothesis”

As discussed above, there are several serious doubts on the validity of the “cholesterol hypothesis” for the etiology of atherosclerosis. Therefore an attempt was made to define a better hypothesis. Based on the evidence discussed above it was considered that an alternative hypothesis had to fulfill the following requirements: 1) HMGCoA-reductase should play a crucial role in the induction or progression of cardiovascular disease; 2) The alternative hypothesis should explain the correlation which exists between plasma cholesterol and the incidence of cardiovascular disease and 3) Plasma-cholesterol should be no causal factor for atherosclerosis.

**Figure 1:**
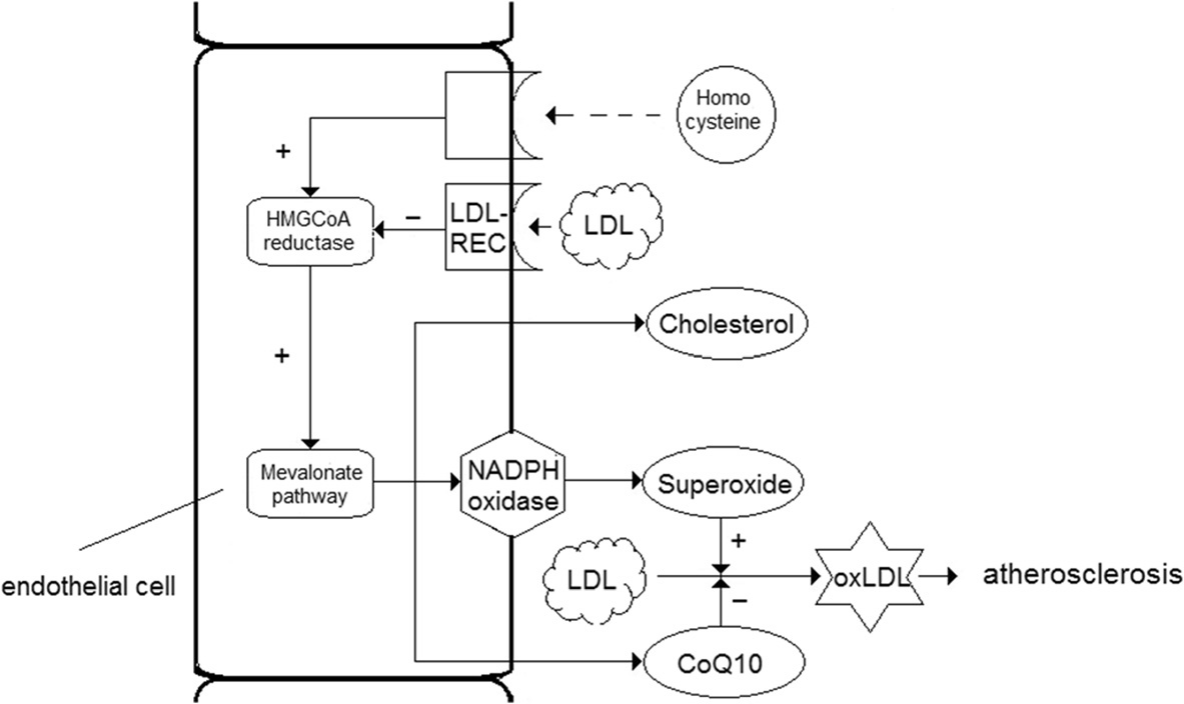
**The mevalonate–hypothesis.** Inflammatory factors like homocysteine can activate the mevalonate pathway. Also reduced uptake of LDL cholesterol (LDL) by LDL receptors (LDL-REC) can activate the mevalonate pathway. The mevalonate pathway regulates numerous biological activities including the formation of coenzyme Q10, the formation of cholesterol and the activation of NADPH oxidase. Activation of NADPH oxidase can contribute to the formation of ox-LDL cholesterol which is probably an important trigger for the induction of atherosclerosis. Coenzyme Q10 plays an important role as an anti-oxidant in the protection of LDL cholesterol from oxidation.

The simplest hypothesis which would fulfill these criteria is the “mevalonate hypothesis” (shown in Figure 1). This hypothesis states that an overactive mevalonate pathway in endothelial cells is probably an important causal factor in atherosclerosis since this would result in the activation of NADPH-oxidase. NADPH oxidase produces superoxide free radicals which transform native LDL cholesterol into ox-LDL, a widely accepted causal factor for atherosclerosis [34].

The “mevalonate hypothesis” fulfills the requirements as listed above: In this hypothesis, activation of HMGCoA reductase results in the formation of free radicals by NADPH-oxidase which transform native LDL into ox-LDL. Since after activation of HMGCoA reductase cholesterol is produced, a correlation between plasma cholesterol and atherosclerosis is ensured. Furthermore in this hypothesis cholesterol-production is not related to causing atherosclerosis.

The “mevalonate hypothesis” followed from the knowledge that this pathway plays a central role in the activation of endothelial NADPH oxidase. This enzyme is activated by the small GTPase Rac1, which for its activity is dependent on isoprenylation, regulated by the mevalonate pathway [35]. NADPH oxidase is the strongest contributor to the formation of free radicals by endothelial cells. Inflammatory factors which activate endothelial NADPH oxidase probably play an important role in atherosclerosis [36]. These endothelial NADPH-oxidase activating and pro-atherogenic factors include at least TNF alpha, angiotensin, homocysteine, interferon gamma, interleukin1, interleukin 6 and interleukin 8. Finally, endothelial activation of NADPH-oxidase is associated with arterial stiffness [37], suggesting a role for this enzyme activity in atherosclerosis.

There is ample experimental evidence to show that activation of HMGCoA reductase results in activation of NADPH-oxidase: Chronic exposure of cells to statins (pharmacological inhibitors of HMGCoA reductase) leads to a major upregulation of HMGCoA reductase activity [38]. After sudden statin withdrawal, a strong increase in the NADPH oxidase dependent production of free radicals was observed in the aorta of mice [39]. A similar effect was seen after statin withdrawal from human endothelial cells in culture [39]. These data confirm that stimulation of the mevalonate pathway can result in the formation of NADPH-oxidase. Chronic treatment with statins also potently up-regulate the HMGCoA reductase activity in humans [40,41]. The clinical consequence of suddenly stopping statin treatment after such up-regulation of HMGCoA reductase is an increase in ischemic stroke [42] and an increased event rate in patients with acute coronary syndromes [43]. These adverse cardiac effects were independent of changes in plasma cholesterol levels. Interestingly, statin withdrawal in humans also resulted in a rebound inflammatory response during the acute phase of myocardial infarction [44] and in endothelial dysfunction [45]. In combination, these data confirm that activation of HMGCoA reductase can be responsible for activation of endothelial NADPH-oxidase and that this can contribute to cardiovascular disease.

There are additional human models in which increased activity of endothelial HMGCoA reductase is observed: In familial hypercholesterolemia, due to a genetic defect, all cells lack functional LDL-receptors which are necessary for cholesterol uptake. Due to a lack of intracellular cholesterol, cells of people with familial hypercholesterolemia have increased plasma cholesterol levels and increased levels of HMGCoA reductase. For fibroblasts this latter effect was quantified [46], but effects on HMGCoA reductase in the same direction are expected in endothelial cells. Cells of people with FH not only have increased levels of HMGCoA reductase, cells in the arterial wall of these patients also show at least a doubling of NADPH-oxidase activity [47], suggesting that an increased mevalonate pathway is responsible for that. As mentioned previously, in these people the incidence of atherosclerosis is clearly increased. These clinical data in patients with FH confirm that an increased activity of the mevalonate pathway can lead to cardiovascular disease.

Further evidence for the theory that increased HMGCoA reductase leads to increased NADPH oxidase activity and cardiovascular disease comes from studies on people with mutant forms of ApoE. Cells of patients with these mutations cannot take up cholesterol since ApoE is required for that. As a consequence of this and as explained above, these cells will have increased HMGCoA reductase activities, and people with this mutation have increased plasma cholesterol values. A direct effect of this mutation of endothelial NADPH oxidase activity was not measured yet in humans, but in animals this mutation indeed leads to increased HMGCoA reductase activity [47]. It is therefore likely that endothelial cells of people with mutant ApoE phenotypes also have an increased activity of the mevalonate pathway. This may explain the increased incidence of cardiovascular disease in patients with these mutant ApoE phenotypes [48].

Not only atherosclerosis as observed in FH or in patients with ApoE mutations is related to an increased activity of HMGCoA reductase. Industrial trans fatty acids, well known for their adverse cardiovascular effects [49], stimulate NADPH oxidase in endothelial cells [50] and can also stimulate endogenous cholesterol synthesis [51]. Therefore, atherosclerosis induced by industrial trans-fatty acids may also be dependent on activation of the mevalonate pathway. Bacterial lipopolysaccharide (LPS) is produced by bacteria in the gut. Diets not only affect the amount and types of LPS produced but also the uptake of LPS from the gut. LPS is likely to contribute to atherosclerosis in humans [52] and circulates in the blood in concentration which are relevant with respect to its pro-inflammatory effects. LPS at relevant concentrations increased endothelial superoxide production in human saphenous veins, which led to superoxide- dependent binding of monocytes to the endothelia. This phenomenon which is seen as a model for early phase atherosclerosis, was inhibited by statins [52], suggesting the involvement of the mevalonate pathway. Together these data imply that diet-induced atherosclerosis may also follow a mechanism covered by the “mevalonate hypothesis”.

The “mevalonate hypothesis” implies that high levels of inflammatory factors can lead to a concomitant increase in cholesterol production and NADPH-oxidase activity. This dual action may have a logical explanation. LPS, the archetype inflammatory factor, is detoxified in vivo by binding to LDL cholesterol [53]. Therefore evolutionary, the production of cholesterol by endothelial cells, the primary targets for LPS toxicity, in response to inflammatory factors appears to make sense. Concomitant activation of NADPH-oxidase is very useful since this has a direct toxic effect against invading bacteria [54].

Protection of plasma cholesterol against bacterial infections is not just theory. Large epidemiological studies show an inverse correlation between the occurrence of infectious disease and plasma cholesterol levels [55,56]. Furthermore, rats made hypolipidaemic by pharmacological tools had a marked increased endotoxin-induced mortality compared to control rats, whereas administration of exogenous lipoprotein reduced their mortality substantially [57].

The liver is known to be the primary organ for the regulation of plasma cholesterol. However since the endothelial cells represent a huge number of cells present throughout the entire body, probably representing the largest “organ” of the body, even a relatively low rate of cholesterol production by these cells can probably be sufficient to increase plasma cholesterol levels under pro-inflammatory conditions. Therefore, under chronic pro-atherogenic (pro-inflammatory) conditions it is quite possible that enhanced production of cholesterol by endothelial cells [58] will contribute to the increased plasma cholesterol levels.

### Implications of the “mevalonate hypothesis”

#### For plasma cholesterol as a biomarker

If the “mevalonate hypothesis” would be correct, plasma cholesterol levels can still be used as a biomarker for cardiovascular disease in populations who are not exposed to cholesterol lowering drugs. However, plasma cholesterol levels of populations treated with plasma cholesterol-modifying drugs or functional foods are not necessarily useful as biomarkers for cardiovascular risk, since not all mechanisms which lead to decreased cholesterol plasma levels will inhibit the mevalonate pathway, and not all mechanisms which lead to increased cholesterol plasma levels will activate the mevalonate pathway.

#### For approval of functional foods by regulatory authorities

Health claims for several functional food ingredients have been approved by EFSA based on their effect on plasma cholesterol only. Most of these ingredients lower plasma cholesterol by lowering cholesterol uptake from the gut. However since reduced uptake of cholesterol (due to lower plasma cholesterol levels) is likely to stimulate HMGCoA reductase, these ingredients may actually worsen cardiovascular risk if the hypothesis of this paper would be correct. Therefore, these approvals may need reconsideration.

## Abbreviations

FH: Familial hypercholesterolemia; LDL: Low density lipoprotein; IDL: Intermediate density lipoprotein; HDL: High density lipoprotein; EFSA: European Food and Safety Authority; LRC-CPPT: The Lipid Research Clinics Coronary Primary Prevention Trial; Ox-LDL: Oxidized LDL cholesterol; HMGCoA reductase: 3-hydroxy-3-methylglutaryl coenzyme A reductase; NADPH oxidase: Nicotinamide adenine dinucleotide phosphate oxidase.

## Competing interests

The author declares not to have any competing interests.

## References

[B1] Steinberg D An interpretive history of the cholesterol controversy: Part I J Lipid Res 2004 45 1583 1593 10.1194/jlr.R400003-JLR200 15102877

[B2] Steinberg D An interpretive history of the cholesterol controversy: Part II: the early evidence linking hypercholesterolemia to coronary disease in humans J Lipid Res 2005 46 179 190 15547293 10.1194/jlr.R400012-JLR200

[B3] Steinberg D An interpretive history of the cholesterol controversy: Part III: mechanistically defining the role of hyperlipidemia J Lipid Res 2005 46 2037 2051 10.1194/jlr.R500010-JLR200 15995167

[B4] Steinberg D An interpretive history of the cholesterol controversy: Part IV:The 1984 coronary primary prevention trial ends it-almost J Lipid Res 2006 47 1 14 16227628 10.1194/jlr.R500014-JLR200

[B5] Steinberg D6 An interpretive history of the cholesterol controversy: PartV: The discovery of the statins and the end of the controversy J Lipid Res 2006 47 1339 1351 10.1194/jlr.R600009-JLR200 16585781

[B6] Wanders AJ Brouwer IA Siebelink E Katan MB Effect of a high intake of conjugated-linoleic acid on lipoprotein levels in healthy humans subjects PLoS One 2010 5 2 E9000 10.1371/journal.pone.0009000 20140250 PMC2815780

[B7] Windaus A Uber den gehalt nirmaler und atheromatoser aorten on cholsterin und cholesterinestern. Hoppe-Seyler’s Z Physiol Chem 1910 67 174 176 10.1515/bchm2.1910.67.2.174

[B8] Anitschkow NN Ueber experimentelle Choleserinsteatose und ihre Bedeutung fur die Entstehung einiger pathologischer Prozesse Zentralbl Allg Pathol 1913 24 1 9

[B9] Muller L Angina pectoris in heriditary xanthomatosis Arch Intern Med 1939 64 675 700 10.1001/archinte.1939.00190040016002

[B10] Gofman JW Lindgren F Elliott H Mantz W Hewitt J Herring V The role of lipids and lipoproteins in atherosclerosis Science 1950 111 166 171 10.1126/science.111.2877.166 15403115

[B11] Barr DP Russ EM Adler HE Protein-lipid relationships in human plasma. II. In atherosclerosis and related conditions Am J Med 1951 11 480 488 10.1016/0002-9343(51)90183-0 14885223

[B12] Brown MS Goldstein JL Familial hypercholesterolemia: defective binding of lipoproteins to cultured fibroblasts associated with impaired regulation of 3-hydroxy-3-methylglutaryl coenzyme A reductase activity Proc Natl Acad Sci USA 1974 71 788 792 10.1073/pnas.71.3.788 4362634 PMC388099

[B13] Fielding CJ Fielding PE Cholesterol transport between cells and body fluids. Role of plasma lipoproteins and the plasma cholesterol esterification system Med Clin North Am 1982 66 363 373 7040842 10.1016/s0025-7125(16)31425-0

[B14] Kannel WB Dawber TR Kagan A Revotskie N Stokes J Factors of risk in the development of coronary heart disease—six year follow-up experience. The Framingham Study Ann Intern Med 1961 55 33 50 13751193 10.7326/0003-4819-55-1-33

[B15] Kinsell LW Partridge J Boling L Margen S Michael G Dietary modification of serum cholesterol and phospholipid levels J Clin Endocrinol Metab 1952 12 909 913 14938428 10.1210/jcem-12-7-909

[B16] Leren P The effect of plasma cholesterol lowering diet in male survivors of myocardial infarction. A controlled clinical trial Acta Med Scand 1966 466 Suppl 1 92 5228820

[B17] Keys Seven Countries: A Multivariate Analysis of Death and Coronary Heart Disease 1980 Cambridge: Harvard University Press ISBN 0-674-80237-3

[B18] Lipid Research Clinics Coronary Primary Prevention Trial The Lipid Research Clinics Coronary Primary Prevention Trial results. II. The relationship of reduction in incidence of coronary heart disease to cholesterol lowering J Am Med Assoc 1984 251 365 374 6361300

[B19] Scandinavian Simvastatin Survival Study Group Randomised trial of cholesterol lowering in 4444 patients with coronary heart disease: the Scandinavian Simvastatin Survival Study (4S) Lancet 1994 344 1383 1389 7968073

[B20] Briel M Ferreira-Gonzalez I You JJ Karanicolas PJ Akl EA Wu P Blechacz B Bassler D Wei X Sharman A Whitt I Alves Da Silva S Khalid Z Nordmann A Zhou Q Walter SD Vale N Bhatnagar N O’Regan C Mills EJ Bucher HC Montori VM Guyatt GH Association between change in high density lipoprotein cholesterol and cardiovascular disease morbidity and mortality: systematic review and meta-regression analysis BMJ 2009 338 b92 10.1136/bmj.b92 19221140 PMC2645847

[B21] Haase L Tybjærg-Hansen A Ali Qayyum A Schou J Nordestgaard BG Frikke-Schmidt R LCAT, HDL Cholesterol and Ischemic Cardiovascular Disease: A Mendelian Randomization Study of HDL Cholesterol in 54,500 Individuals J Clin Endocrinol Metab 2012 97 2 E248 E256 10.1210/jc.2011-1846 22090275

[B22] Hulley S Grady D Bush T Furberg C Herrington D Riggs B Vittinghoff E Randomized Trial of Estrogen Plus Progestin for Secondary Prevention of Coronary Heart Disease in Postmenopausal Women JAMA 1998 280 605 13 10.1001/jama.280.7.605 9718051

[B23] Kastelein JJP Van Leuven SI Burgess L Evans GW Kuivenhoven JA Barter PJ Revkin JH Grobbee DE Riley WA Shear CL Duggan WT Bots ML For the RADIANCE 1 Investigators: Effect of Torcetrapib on Carotid Atherosclerosis N Engl J Med 2007 356 1620 1630 10.1056/NEJMoa071359 17387131

[B24] Kastelein JJP Akdim F Stroes ESG Zwinderman AH Bots ML Stalenhoef AFH Frank FCRP Visseren LJ Sijbrands EJG Trip MD Stein EA Gaudet D Duivenvoorden R Veltri EP Marais AD De Groot E for the ENHANCE Investigators: Simvastatin with or without Ezetimibe in Familial Hypercholesterolemia N Engl J Med 2008 358 1431 1443 10.1056/NEJMoa0800742 18376000

[B25] Beng C Fellström MD Jardine AG Roland E Schmieder MD Hallvard Holdaas MD For the AURORA Study Group: Rosuvastatin and Cardiovascular Events in Patients Undergoing Hemodialysis N Engl J Med 2009 360 1395 1407 10.1056/NEJMoa0810177 19332456

[B26] Knopp RH d’Emden M Smilde JG Pocock SJ on behalf of the ASPEN study group Efficacy and safety of atorvastatin in the prevention of cardiovascular endpoints in subjects with type 2 diabetes. The Atorvastatin study for prevention of coronary heart disease endpoints in non-insulin dependent diabetes mellitus (ASPEN) Diabetes Care 2006 29 7 1478 1485 10.2337/dc05-2415 16801565

[B27] Wanner C Krane V Marz W Olschewski M Mann JFE Ruf G Ritz E Atorvastatin in patients with type 2 diabetes mellitus undergoing hemodialysis N Engl J Med 2005 353 238 248 10.1056/NEJMoa043545 16034009

[B28] Tavazzi L Maggioni AP Marchioli R The Gissi-HF investigators: Effect of Rosuvastatin in patients with chronic heart failure (the GISSI-HF trial): a randomised, double-blind, placebo-controlled trial Lancet 2008 372 1231 12 10.1016/S0140-6736(08)61240-4 18757089

[B29] Langsjoen PH Langsjoen AM The clinical use of HMGCoA reductase inhibitors and the associated depletion of coenzyme Q10. A review of animal and human publications Biofactors 2003 18 101 111 10.1002/biof.5520180212 14695925

[B30] Mohr D Bowry V Stocker R Dietary supplementation with coenzyme Q10 results in increased levels of ubiquinol-10 within circulating lipoproteins and increased resistance of human low-density lipoprotein to the initiation of lipid peroxidation Biochim Biophys Acta 1992 1126 247 254 10.1016/0005-2760(92)90237-P 1637852

[B31] Folkers K Vadhanavikit S Mortensen SA Biochemical rationale and myocardial tissue data on the effective therapy of cardiomyopathy with coenzyme Q10 PNAS 1985 82 901 904 10.1073/pnas.82.3.901 3856239 PMC397155

[B32] Miettinen T Gylling H Baseline serum cholestanol as predictor of recurrent coronary events in subgroup of Scandinavian simvastatin survival study BMJ 1998 316 1127 10.1136/bmj.316.7138.1127 9552949 PMC28514

[B33] Miettinen TA Strandberg TE Gylling H for the Finnish Investigators of the Scandinavian Simvastatin Survival Study Group Noncholesterol sterols and cholesterol lowering by long-term simvastatin treatment in coronary patients. Relation to basal serum cholestanol Arterioscler Thromb Vasc Biol 2000 20 1340 1346 10.1161/01.ATV.20.5.1340 10807752

[B34] Stocker R Keaney JF Role of oxidative modifications in atherosclerosis Physol Rev 2004 84 4 1381 1478 10.1152/physrev.00047.2003 15383655

[B35] Gregg D Rauscher FM Goldschmidt-Clermont PJ Rac regulates cardiovascular superoxide through diverse molecular interactions: more than a binary GTP switch Am J Physiol Cell Physiol 2003 285 4 C723 C734 12958025 10.1152/ajpcell.00230.2003

[B36] Rueckschloss U NADPH Oxidase in Endothelial Cells: Impact on Atherosclerosis Antioxid Redox Signal 2004 5 2 171 180 12716477 10.1089/152308603764816532

[B37] de Oliveira Alvim R Lima Santos PC Jr Gonçalves Dias R Velho Rodrigues M de Sa Cunha R Mill JG Nadruz W Jr Krieger JE Costa Pereira A Association between the C242T polymorphism in the p22phox gene with arterial stiffness in the Brazilian population Physiol Genomics 2012 44 10 587 592 10.1152/physiolgenomics.00122.2011 22496489

[B38] Brown MS Goldstein JL Multivalent feedback regulation of HMG CoA reductase, a control mechanism coordinating isoprenoid synthesis and cell growth J Lipid Res 1980 21 505 517 6995544

[B39] Vecchione C Brandes RP Withdrawel of 3-hydroxy-3-methylglutaryl Coenzyme A reductase inhibitors elicit oxidative stress and induces endothelial dysfunction in mice Circulation res 2002 91 173 179 10.1161/01.RES.0000028004.76218.B8 12142351

[B40] Parini P Gustafsson U Davis MA Larsson L Einarsson C Wilson M Rudling M Tomoda H Omura S Cholesterol synthesis inhibition elicits an integrated molecular response in human livers including decreased ACAT2 Arterioscler Thromb Vasc Biol 2008 28 1200 1206 10.1161/ATVBAHA.107.157172 18340009 PMC2757773

[B41] Reihner E Rudling M Stahlberg D Berglund L Ewerth S Bjorkhem I Einarsson K Angelin B Influence of pravastatin, a specific inhibitor of HMG-CoA reductase, on hepatic metabolism of cholesterol N Engl J Med 1990 323 224 8 10.1056/NEJM199007263230403 2114543

[B42] Blanco M Nombela F Castellanos M Rodriguez-Yanez M Garcia-Gil M Leira R Lizasoain I Serena J Vivancos J Statin treatment withdrawal in ischemic stroke: a controlled randomized study Neurology 2007 69 904 10 10.1212/01.wnl.0000269789.09277.47 17724294

[B43] Heeschen C Hamm CW Laufs U Snapinn S Bohm M White HD Withdrawal of statins increases event rates in patients with acute coronary syndromes Circulation 2002 105 1446 1452 10.1161/01.CIR.0000012530.68333.C8 11914253

[B44] Sposito AC Carvalho LS Cintra RM Araujo AL Ono AH Andrade JM Coelho OR Silva JC Qe Rebound inflammatory response during the acute phase of myocardial infarction after simvastatin withdrawal Atherosclerosis 2009 207 191 194 10.1016/j.atherosclerosis.2009.04.008 19464010

[B45] Chen H Ren JY Xing Y Zhang WL Liu X Wu P Wang RJ Luo Y Short-term withdrawal of simvastatin induces endothelial dysfunction in patients with coronary artery disease: a dose-response effect dependent on endothelial nitric oxide synthase Int J Cardiol 2009 131 313 320 10.1016/j.ijcard.2007.10.044 18221806

[B46] Goldstein JL Brown MS Familial Hypercholesterolemia: Identification of a Defect in the Regulation of 3-Hydroxy-3-Methylglutaryl Coenzyme A Reductase Activity Associated with Overproduction of Cholesterol PNAS 1973 70 10 2804 2808 10.1073/pnas.70.10.2804 4355366 PMC427113

[B47] Martino F Loffredo L Carnevali R Sanguigni V Martino E Catasca E Zanoni C Pignatelli P Violi F Oxidative Stress Is Associated With Arterial Dysfunction and Enhanced Intima-Media Thickness in Children With Hypercholesterolemia: The Potential Role of Nicotinamide-Adenine Dinucleotide Phosphate Oxidase Pediatrics 2008 122 3 e648 e655 10.1542/peds.2008-0735 18762499

[B48] Song Y Stampfer MJ Liu S Meta-analysis: apolipoprotein E genotypes and risk for coronary heart disease Ann Intern Med 2004 141 2 137 47 15262670 10.7326/0003-4819-141-2-200407200-00013

[B49] Mozaffarian D Katan MB Ascherio A Stampfer MJ Willett WC Trans fatty acids and cardiovascular disease N Engl J Med 2006 354 1601 1613 10.1056/NEJMra054035 16611951

[B50] Bryk D Zapolska-Downar D Melecki M Hajdukiewicz D Sitkiewicz D Trans-fatty acids induce a proinflammatory response in endothelial cells through ROS-dependent nuclear factor Kappa B activation J Phys Pharmacol 2011 62 2 229 238 21673371

[B51] Sundram K French MA Clandinin MT Exchanging partially hydrogenated fat for palmitic acid in the diet increases LDL-cholesterol and endogenous cholesterol synthesis in normocholesterolemic women Eur J Nutr 2003 42 188 94 10.1007/s00394-003-0411-9 12923649

[B52] Rice JB Stoll LL Li W-G Denning GM Weydert J Charipar E Richenbacher WE Miller FJ Jr Weintraub NL Low level endotoxin induces potent inflammatory activation of human blood vessels Arterioscl Throm Vasc Biol 2003 23 1576 1582 10.1161/01.ATV.0000081741.38087.F9 12816876

[B53] Ravnskov U High cholesterol may protect against infections and atherosclerosis Q J Med 2003 96 927 934 10.1093/qjmed/hcg150 14631060

[B54] De Assis MC Da Costa AO Barja-Fidalgo TC Plotkowsk MC Human endothelial cells are activated by interferon-γ plus tumour necrosis factor-α to kill intracellular Pseudomonas aeruginosa Immunology 2000 101 2 271 278 10.1046/j.1365-2567.2000.00102.x 11012781 PMC2327068

[B55] Iribarren C Jacobs DR Jr Sidney S Claxton AJ Gross MD Sadler M Blackburn H Serum total cholesterol and risk of hospitalization and death from respiratory disease Int J Epidemiol 1997 26 1191 1202 10.1093/ije/26.6.1191 9447398

[B56] Iribarren C Jacobs DR Jr Sidney S Claxton AJ Feingold KR Cohort study of serum total cholesterol and in-hospital incidence of infectious diseases Epidemiol Infect 1998 121 335 347 10.1017/S0950268898001435 9825784 PMC2809530

[B57] Feingold KR Funk JL Moser AH Shigenaga JK Rapp JH Grunfeld C Role of circulating lipoproteins in protection from endotoxin toxicity Infect Immun 1995 63 2041 2046 7729918 10.1128/iai.63.5.2041-2046.1995 PMC173262

[B58] Li H Lewis A Brodsky S Rieger R Iden C Goligorsky MS Homocysteine Induces 3-Hydroxy-3-Methylglutaryl Coenzyme A Reductase in Vascular Endothelial Cells. A Mechanism for Development of Atherosclerosis? Circ 2002 105 1037 1043 10.1161/hc0902.104713 11877351

